# Impact of Depression in Sickle Cell Disease Hospitalization-Related Outcomes: An Analysis of the National Inpatient Sample (NIS)

**DOI:** 10.3390/medicina55070385

**Published:** 2019-07-17

**Authors:** Henry K. Onyeaka, Uwandu Queeneth, Wahida Rashid, Naveed Ahmad, Shanthini Kuduva Rajan, Paul Rahul Jaladi, Rikinkumar S. Patel

**Affiliations:** 1 Harvard School of Public Health, Boston, MA 02115, USA; 2 Department of Psychiatry, Maastricht University, 4–6, 6211 LK Maastricht, The Netherlands; 3 Department of Medicine, Dhaka Medical College, Dhaka 1000, Bangladesh; 4 Department of Psychiatry, University of Texas, Houston, TX 77021, USA; 5 Tirunelveli Medical College, Tirunelveli 627011, India; 6 Rajiv Gandhi Institute of Medical Sciences, Kadapa 516002, India; 7 Department of Psychiatry, Griffin Memorial Hospital, Norman, OK 73071, USA

**Keywords:** sickle cell disease, depression, hospitalization outcomes, demographics, morbidity, mortality

## Abstract

*Background and objectives*: This study aimed to analyze and discern the differences in demographics and inpatient outcomes (length of stay (LOS), total charges, disease severity, and mortality) between depressed versus non-depressed sickle cell disease (SCD) patients. *Materials and Methods*: A retrospective analysis was conducted using the Nationwide Inpatient Sample (2010–2014). We identified 73,225 SCD hospitalizations and comorbid depression (6317, 8.6%) as the primary and the other diagnosis, respectively, using International Classification of Diseases (ICD)-9 codes. We used linear and logistic regression model to evaluate the changes in inpatient outcomes. *Results*: Comorbid depression was more prevalent among middle-aged adults (11.5%), females (10.63%), and whites (12.43%). We did not find any association between income and comorbid depression among SCD patients. After adjusting for the demographic covariates, comorbid depression remained a significant risk factor for longer LOS (mean difference −1.16 days, 95% CI −1.30 to −1.03) and higher total charges (mean difference −USD5058, 95% CI −6261 to −3855) during hospitalization. SCD with comorbid depression was also significantly associated with a higher number of chronic conditions (mean difference −2.08, 95% CI −2.13 to −2.03) and 1.5 times (95% CI 1.39 to 1.63) higher odds of major severity of illness. *Conclusion*: Comorbid depression was significantly associated with longer LOS, more severity of illness, and higher hospital charges. Healthcare providers caring for adults with SCD should consider screening for and treating comorbid depression to improve the health-related quality of life.

## 1. Introduction

Sickle cell disease (SCD) is the most common genetic hemoglobin disorder in the US and affects about 100,000 Americans, primarily those of African and Mediterranean descent [1]. It is a chronic and debilitating disease that causes a significant amount of economic and health burden for those affected. Because it impacts a variety of organ systems, several complications are common among SCD patients, including chronic leg ulcers, strokes, retinopathy, anemia, osteonecrosis, acute chest syndrome, cholelithiasis, as well as bacterial infections due to autosplenectomy [2]. The chronic nature of the disease and its numerous complications contribute to the impairment of physical and mental states over time, resulting in a significantly lower health-related quality of life [3,4].

Advances and evolution in medicine have significantly improved the survival and quality of life for SCD patients [5]. SCD was once associated with early childhood mortality, but today in the United States, more than 90% of people with SCD live into adulthood [6]. Despite these advances, adult SCD patients face many challenges of living with a chronic condition that necessitates lifelong medical management that may predispose them to the risk of psychiatric illnesses, including depressive and anxiety disorders [7,8,9]. Comorbid depression is associated with worse adverse events and outcomes. Jerrell et al. found that SCD patients with depression had a higher prevalence of acute vaso-occlusive pain and acute chest syndrome visits per year, developed more complications related to organ damage, and incurred significantly higher outpatient and inpatient total healthcare costs compared to controls [9]. About 51.8% of the healthcare costs of these patients were directly related to SCD, and about 80% of these SCD-related healthcare costs were associated with inpatient hospitalizations [10] The estimated total health care cost per year for children and adults with SCD in the US was estimated at USD1.1 billion [10].

Furthermore, depression among patients with SCD is known to be associated with a higher rate of healthcare utilization and, consequently, higher costs of medical care [11,12,13]. Adam et al. found that health care costs for adult SCD patients with depression were more than double those of SCD patients without depression [14]. So far, these studies have been limited by their relatively small sample size. Also, less is known about the impact of co-morbid depression on morbidity and mortality in a nationally representative sample of hospitalized adult SCD patients.

Therefore, we utilized the nationwide inpatient sample (NIS) data to discern the differences in demographic characteristics between SCD patients with versus without comorbid depression. Next, we evaluated the odds of major severity of illness and mortality in SCD patients with depression and the change in length of stay (LOS) and total charges during hospitalization compared to the non-depressed group.

## 2. Materials and Methods

### 2.1. Data Source

A retrospective analysis was conducted using the NIS data from 2010 to 2014 [15]. The NIS is one of the largest inpatient databases from the Healthcare Cost and Utilization Project (HCUP) that covers about 4411 hospitals and 45 states in the United States. To protect the privacy of patients, physicians, and hospitals, the state and hospital identifiers are de-identified [15]. We were not required to obtain Institutional Review Board permission, as this study utilized the publicly available de-identified data.

### 2.2. Inclusion Criteria

We included adult inpatients ≥18 years of age with a principal diagnosis of SCD (*N* = 73,225) by identifying the International Classification of Diseases (ICD)-9 codes 282.41, 282.42, 282.60–282.64, 282.68, or 282.69 in diagnoses field DX1 in the NIS. From the study population, we then identified individuals with comorbid depression (*N* = 6317) by identifying the co-diagnoses fields DX2 to DX25 for ICD-9 codes of 300.4, 301.12, 309.00, 309.1, or 311 [16].

### 2.3. Variables of Interest

Demographic variables evaluated in this study were age, sex, race, median household income, and primary payer [17]. To measure the differences in outcomes in SCD inpatients by comorbid depression, the following variables were included: severity of illness, number of chronic conditions, LOS, total charges, and in-hospital mortality [17]. In the NIS, the severity of illness measures the loss of body functions, and in-hospital mortality is the number of deaths during hospitalization and is all-cause. The LOS refers to the number of nights the patient remained in the hospital for the principal diagnosis (SCD), and total charges during hospitalization do not include professional fees and non-covered charges [17].

### 2.4. Statistical Analyses

We used descriptive statistics for categorical data and independent sample *t*-test for continuous data to measure the differences in demographics and hospital outcomes between depressed and non-depressed SCD inpatients. We applied discharge weight (DISCWT) from the NIS to obtain a national representation of our sample population [17]. We used a binomial logistic regression model to evaluate the odds ratio (OR) for major or extreme severity of illness and in-hospital mortality in depressed SCD inpatients compared to the non-depressed group, and the model was adjusted for and age, sex, race, income, and primary payer. A *p* value < 0.05 was used to determine the statistical significance of the test results. All statistical analyses were conducted using SPSS version 25 (IBM Corp., Armonk, NY, USA).

## 3. Results

### 3.1. Demographic Characteristics

Between 2010 and 2014, a total of 73,225 SCD hospital admissions were included in our study using the NIS database. Of these inpatients, 6317 (8.63%) had comorbid depression. A higher proportion of comorbid depression was seen in middle-aged adults with SCD (35–50 years, 11.5%) and among females (10.63%). Although the majority of the inpatients with SCD were Blacks, comorbid depression was more prevalent among whites (12.43%; 95/764) compared to Blacks (8.65%; 5925/68,484). Also, we found no association between level of income and comorbid depression. The demographic distribution of SCD with comorbid depression is shown in Table 1.

### 3.2. Differences in Inpatients Outcomes

Table 2 displays the adjusted odds ratios (ORs) and mean differences (MDs) for the inpatient outcomes comparing SCD with comorbid depression vs. without comorbid depression. After adjusting for the demographic covariates (age, race, gender, and household income), co-morbid depression remained a significant risk factor for longer length of in-hospital stay (mean difference −1.16, 95% CI −1.30, −1.03; *p* < 0.001). SCD patients with co-morbid depression also had a statistically significant higher total cost of in-patient charges. (mean difference −USD5058, 95% CI −6261, −$3855; *p* < 0.001). We did not find any statistically significant association between SCD with comorbid depression and in-patient mortality (OR 0.59, 95% CI 0.34−1.02; *p* = 0.06). Having SCD with co-morbid depression was significantly associated with a higher number of chronic conditions (mean difference −2.08, 95% CI −2.13, −2.03; *p* < 0.001). Furthermore, compared to SCD alone, SCD with co-morbid depression resulted in a significantly higher likelihood of having major severity of illness and loss of bodily function (OR 1.51, 95% CI 1.39,1.63; *p* < 0.001).

## 4. Discussion

Our study aimed to assess the prevalence of comorbid depression among hospitalized adults with SCD, identify the demographic factors related to comorbid depression, and evaluate the differences in hospitalization outcomes. We found that middle-aged adults (35 to 50 years), female gender, and low socioeconomic status were significantly associated with higher odds of comorbid depression. SCD with comorbid depression was associated with 1.5-folds higher odds for major severity of illness and higher hospital total charges by USD 5058 per admission for SCD when compared to those without depression.

Depression is significantly associated with several chronic diseases [18,19,20,21,22]. As with most chronic diseases, our study shows that comorbid depression is prevalent among adult patients with SCD. This correlates with prior studies showing a high prevalence of comorbid depression among US adults with SCD. In a study by Levenson et al. that included 232 adult SCD patients, 27.6% had comorbid depression [4]. In another cross-sectional study of African-American adults with SCD, 26% reported comorbid depression [23]. In our study, 8.63% (6317/73,225) of SCD patients had comorbid depression. Although our study involved only an inpatient adult population with SCD, the prevalence of comorbid depression among SCD inpatients was higher than the national prevalence of depression (7.1%) among US adults [24]. We observed that 11.5% of middle-aged adults (1895/16,526 patients, OR 1.65, *p* = 0.004) had depression. Also, when compared to elderly patients above 65 years, middle-aged adults had a higher likelihood of comorbid depression.

From our study data, 10.63% (4220/39,681) of all SCD patients with comorbid depression were females, and they had 1.77-fold higher odds for comorbid depression then males. Again, this is consistent with the 1.5-fold higher national prevalent rates for depression among US adults when comparing females to males [1,24]. Although some studies have evaluated the association between SCD and comorbid depression, there is a dearth of literature relating to gender differences among adult SCD patients with comorbid depression. There are a few studies which show no significant association between gender and comorbid depression among SCD patients [25,26,27]. However, our results are consistent with findings from other studies, which also reported that comorbid depression was significantly higher among females with SCD compared to males [12,14,28].

Furthermore, we found no association between SCD with comorbid depression and income level. This is consistent with observations in other studies. For example, Adam et al. did not find any differences due to socioeconomic status [14]. Additionally, we observed that the LOS and total charges during SCD hospitalization were much higher in those with comorbid depression. Our study shows that comorbid depression was associated with an increase in hospitalization cost by USD5,058 per admission. In a previous study that reported similar findings, Adam et al. observed that total average healthcare costs (including inpatient, emergency visit, and outpatient costs) were higher in the depression group, both before and after assessment of depression [14]. The bulk of this cost was attributed to the cost of inpatient care. They observed that inpatient costs were significantly higher in the depressed group in the year preceding enrollment, despite a non-significant difference in the number of inpatient days. Additionally, studies in adolescents and children with SCD and comorbid depression populations have also reported significant differences in inpatient LOS [29,30].

The lifespan of individuals living with SCD continues to increase, and most affected individuals in developed countries now live into adulthood. Several factors have contributed to this increased survival among SCD patients. The use of prophylactic penicillin as well as vaccinations against infections have played a role in the improved longevity among people with SCD [31,32,33]. Yet, the transition from pediatric to adult care remains challenging, since the focus of care differs. Also, SCD patients and other underserved minorities often have barriers of access to psychiatrists. Cultural stigma and social determinants of health can impede care for depression. Sickle cell specialists are mostly hematologists who might not feel comfortable treating depression. As a result, care for patients with SCD often falls to primary care providers who may not be fully aware of the many challenges and issues faced by patients and the current management strategies for SCD.

The results of our study indicate that physicians and healthcare providers should actively screen for psychiatric comorbidities like depression in patients with SCD. A biopsychosocial approach to the management of SCD that includes collaborative psychiatric care remains crucial in improving the health outcomes of these patients. Although some multidisciplinary models exist for the management of chronic pain in SCD [34], few centers offer collaborative models to manage SCD. In a report by the National Heart, Lung, and Blood Institute in 2014 to guide the management of SCD, most of the recommendations made covered all but the psychiatric complications that were common among people with SCD [35]. Novel approaches to care of depression can be adopted in the care of SCD. There is evidence that mobile health technologies [13], electronic-health [36], faith-based [37], and community-based therapies [38] can improve patient outcomes.

However, the results in our study should be interpreted with some limitations in mind. Firstly, utilization of inpatient data (and not patient-level data) as the unit of analysis does not translate to generalizability for all patients with SCD. Secondly, this study failed to evaluate other important characteristics, such as patients’ complications and comorbidities, which may be associated with depression. It should be also noted that this is a cross-sectional study that did not evaluate the causal association between depression and SCD. Furthermore, coding errors may have influenced our results, since these errors tend to be more prevalent when administrative datasets such as the NIS are used to analyze SCD and other rare diseases in the US. In addition, these results do not indicate causation. Depression could be a consequence of severe sickle cell disease, and the associations with longer lengths of stay could be due to greater severity of problems. Therefore, we recommend that future research should evaluate the influence of comorbid depression in SCD with clinical data.

One of the significant strengths of our study is that we used the NIS as a nationally representative sample of patients diagnosed with SCD and comorbid depression. The large sample size we obtained ensures that our study is high powered to discern any differences that exist between SCD patients with vs. without comorbid depression. Lastly, since we applied sampling weights to generate estimates of inpatient outcomes, our results are generalizable to a much large population than the sample studied.

## 5. Conclusions

Among hospitalized patients with SCD, comorbid depression is associated with severe morbidity with loss of body functioning, longer hospitalization stays, and higher total charges compared to the inpatients without depression. Integrating the care of adults with SCD among primary care physicians and psychiatrists can improve health outcomes and reduce the cost and burden of illness associated with the disease. Improved awareness of the risks for psychiatric disorders like depression among SCD patients could lead to earlier diagnosis and better management. Therefore, care of SCD patients that utilizes a collaborative and multidisciplinary model remains crucial in improving their health-related quality of life.

## Figures and Tables

**Table 1:** Demographic characteristics in sickle cell disease hospitalizations by comorbid depression.

Variable	Depression		Logistic Regression Model		
	No (%)	Yes (%)	Odds Ratio	95% Confidence Interval	*p* Value
Total inpatients	66,908	6317	-	-	-
**Age at Admission, in Years**					
18–34	68.9	62.9	1.39	0.99–1.96	0.055
35–50	24.7	30.0	1.65	1.17–2.33	0.004
51–64	5.7	6.4	1.41	0.99–2.01	0.058
65+	0.7	0.6	Reference		
**Sex**					
Male	47.0	33.2	Reference		
Female	53.0	66.8	1.77	1.67–1.88	<0.001
**Race**					
White	1.0	1.5	Reference		
Black	93.5	93.8	0.65	0.52–0.82	<0.001
Hispanic	3.1	2.9	0.61	0.46–0.81	0.001
Other	2.3	1.8	0.50	0.37–0.68	<0.001
**Median Household Income, in Percentiles**					
0–25th	49.6	49.4	Reference		
26th–50th	22.6	23.9	1.03	0.97–1.11	0.337
51st–75th	17.4	16.8	0.99	0.92–1.07	0.892
76th–100th	10.3	9.9	0.97	0.88–1.07	0.522

**Table 2:** Inpatient outcomes in sickle cell disease hospitalizations by comorbid depression.

Variable	Depression		Logistic Regression Model		
	No (%)	Yes (%)	Odds Ratio	95% Confidence Interval	*p* Value
Total inpatients	66,908	6317	-	-	-
**Severity of Illness, in Body Loss Functions**					
Minor	38.3	30.5	Reference		
Moderate	43.5	46.2	1.29	1.22–1.38	<0.001
Major	18.3	23.3	1.51	1.39–1.63	<0.001
**Inpatient Mortality**					
Inpatient deaths	0.3	0.3	0.59	0.34–1.02	0.060
No inpatient deaths	99.7	99.7	Reference		
**Other Outcomes**					
Outcome	Mean (SD)	Mean (SD)	Mean Difference	95% CI	*p* value
Number of chronic conditions	3.2 (2.02)	5.3 (2.29)	−2.08	−2.13 to –2.03	<0.001
Length of stay, days	5.3 (5.27)	6.5 (5.89)	−1.16	−1.30 to –1.03	<0.001
Total charges, USD	28,705 (46,633)	33,763 (41,273)	−5058	−6261 to –3855	<0.001

## References

[1] Centers for Disease Control and Prevention Data & Statistics on Sickle Cell Disease. https://www.cdc.gov/ncbddd/sicklecell/data.html.

[2] Bartolucci P., Lionnet F. (2014). Chronic complications of sickle cell disease. Rev. Prat..

[3] Dampier C., LeBeau P., Rhee S., Lieff S., Kesler K., Ballas S., Rogers Z., Wang W. (2011). Health-related quality of life in adults with sickle cell disease (SCD): A report from the comprehensive sickle cell centers clinical trial consortium. Am. J. Hematol..

[4] Levenson J.L., McClish D.K., Dahman B.A., Bovbjerg V.E., Citero V.D.A., Penberthy L.T., Aisiku I.P., Roberts J.D., Roseff S.D., Smith W.R. (2008). Depression and anxiety in adults with sickle cell disease: The PiSCES project. Psychosom. Med..

[5] Quinn C.T., Rogers Z.R., McCavit T.L., Buchanan G.R. (2010). Improved survival of children and adolescents with sickle cell disease. Blood.

[6] American Society of Hematology (2016). State of Sickle Cell Disease 2016 Report.

[7] Benton T.D., Boyd R., Ifeagwu J., Feldtmose E., Smith-Whitley K. (2011). Psychiatric Diagnosis in Adolescents With Sickle Cell Disease: A Preliminary Report. Curr. Psychiatry Rep..

[8] Graves J.K., Hodge C., Jacob E. (2016). Depression, Anxiety, and Quality of Life In Children and Adolescents With Sickle Cell Disease. Pediatr. Nurs..

[9] Jerrell J.M., Tripathi A., McIntyre R.S. (2011). Prevalence and treatment of depression in children and adolescents with sickle cell disease: A retrospective cohort study. Prim. Care Companion CNS Disord..

[10] Kauf T.L., Coates T.D., Huazhi L., Mody-Patel N., Hartzema A.G. (2009). The cost of health care for children and adults with sickle cell disease. Am. J. Hematol..

[11] Carroll P.C., Haywood C., Hoot M.R., Lanzkron S. (2013). A preliminary study of psychiatric, familial, and medical characteristics of high-utilizing sickle cell disease patients. Clin. J. Pain.

[12] Hasan S.P., Hashmi S., Alhassen M., Lawson W., Castro O. (2003). Depression in sickle cell disease. J. Natl. Med. Assoc..

[13] Jonassaint C.R., Kang C., Prussien K.V., Yarboi J., Sanger M.S., Wilson J.D., De Castro L., Shah N., Sarkar U. (2018). Feasibility of implementing mobile technology-delivered mental health treatment in routine adult sickle cell disease care. Transl. Behav. Med..

[14] Adam S.S., Flahiff C.M., Kamble S., Telen M.J., Reed S.D., De Castro L.M. (2017). Depression, quality of life, and medical resource utilization in sickle cell disease. Blood Adv..

[15] (HCUP) Healthcare Cost and Utilization Project Overview of the National (Nationwide) Inpatient Sample (NIS). https://www.hcup-us.ahrq.gov/nisoverview.jsp.

[16] Patel R.S., Shrestha S., Saeed H., Raveendranathan S., Isidahome E.E., Ravat V., Fakorede M.O., Patel V. (2018). Comorbidities and Consequences in Hospitalized Heart Failure Patients with Depression. Cureus.

[17] (HCUP) Healthcare Cost and Utilization Project NIS Description of Data Elements. https://www.hcup-us.ahrq.gov/db/nation/nis/nisdde.jsp.

[18] Bădescu S.V., Tătaru C., Kobylinska L., Georgescu E.L., Zahiu D.M., Zăgrean A.M., Zăgrean L. (2016). The association between Diabetes mellitus and Depression. J. Med. Life.

[19] Newhouse A., Jiang W. (2014). Heart failure and depression. Heart Fail. Clin..

[20] Seligman F., Nemeroff C.B. (2015). The interface of depression and cardiovascular disease: Therapeutic implications. Ann. N. Y. Acad. Sci..

[21] Semenkovich K., Brown M.E., Svrakic D.M., Lustman P.J. (2015). Depression in type 2 diabetes mellitus: Prevalence, impact, and treatment. Drugs.

[22] Yohannes A.M., Alexopoulos G.S. (2014). Depression and anxiety in patients with COPD. Eur. Respir. Rev..

[23] Jenerette C., Funk M., Murdaugh C. (2005). Sickle cell disease: A stigmatizing condition that may lead to depression. Issues Ment. Health Nurs..

[24] NIMH (National Institute of Mental Health) Major Depression.

[25] Alhomoud M.A., Gosadi I.M., Wahbi H.A. (2018). Depression among Sickle Cell Anemia Patients in the Eastern Province of Saudi Arabia. Saudi J. Med. Med Sci..

[26] Alsubaie S.S., Almathami M.A., Abouelyazid A., Alqahtani M.M. (2018). Prevalence of depression among adults with sickle cell disease in the southern region of Saudi Arabia. Pak. J. Med Sci..

[27] Anim M.T., Osafo J., Yirdong F. (2016). Prevalence of psychological symptoms among adults with sickle cell disease in Korle-Bu Teaching Hospital, Ghana. BMC Psychol..

[28] Wison Schaeffer J.J., Gil K.M., Burchinal M., Kramer K.D., Nash K.B., Orringer E., Strayhorn D. (1999). Depression, disease severity, and sickle cell disease. J. Behav. Med..

[29] Myrvik M.P., Burks L.M., Hoffman R.G., Dasgupta M., Panepinto J.A. (2013). Mental health disorders influence admission rates for pain in children with sickle cell disease. Pediatr. Blood Cancer.

[30] Myrvik M.P., Campbell A.D., Davis M.M., Butcher J.L. (2012). Impact of psychiatric diagnoses on hospital length of stay in children with sickle cell anemia. Pediatr. Blood Cancer.

[31] Adamkiewicz T.V., Silk B.J., Howgate J., Baughman W., Strayhorn G., Sullivan K., Farley M.M. (2008). Effectiveness of the 7-valent pneumococcal conjugate vaccine in children with sickle cell disease in the first decade of life. Pediatrics.

[32] Gaston M.H., Verter J.I., Woods G., Pegelow C., Kelleher J., Presbury G., Zarkowsky H., Vichinsky E., Iyer R., Lobel J.S. (1986). Prophylaxis with oral penicillin in children with sickle cell anemia. A randomized trial. N. Engl. J. Med..

[33] Steinberg M.H., Barton F., Castro O., Pegelow C.H., Ballas S.K., Kutlar A., Orringer E., Bellevue R., Olivieri N., Eckman J. (2003). Effect of hydroxyurea on mortality and morbidity in adult sickle cell anemia: Risks and benefits up to 9 years of treatment. Jama.

[34] Taylor L.E.V., Stotts N.A., Humphreys J., Treadwell M.J., Miaskowski C. (2013). A biopsychosocial-spiritual model of chronic pain in adults with sickle cell disease. Pain Manag. Nurs..

[35] National Heart, Lung and Blood Institute (2014). Evidence Based Management of Sickle Cell Disease.

[36] Massoudi B., Holvast F., Bockting C.L., Burger H., Blanker M.H. (2019). The effectiveness and cost-effectiveness of e-health interventions for depression and anxiety in primary care: A systematic review and meta-analysis. J. Affect. Disord..

[37] Warber S.L., Ingerman S., Moura V.L., Wunder J., Northrop A., Gillespie B.W., Rubenfire M. (2011). Healing the heart: A randomized pilot study of a spiritual retreat for depression in acute coronary syndrome patients. Explore J. Sci. Heal..

[38] Stanley M.A., Wilson N., Shrestha S., Amspoker A.B., Armento M., Cummings J.P., Kunik M.E. (2016). Calmer Life: A Culturally Tailored Intervention for Anxiety in Underserved Older Adults. Am. J. Geriatr. Psychiatry.

